# Potential metabolic mechanism of girls' central precocious puberty: a network analysis on urine metabonomics data

**DOI:** 10.1186/1752-0509-6-S3-S19

**Published:** 2012-12-17

**Authors:** Linlin Yang, Kailin Tang, Ying Qi, Hao Ye, Wenlian Chen, Yongyu Zhang, Zhiwei Cao

**Affiliations:** 1 School of Life Science and Technology, Tongji University, Shanghai 200092, China; 2 Shanghai Center for Bioinformation Technology, Shanghai 200235, China; 3 Shanghai University of Traditional Chinese Medicine, Shanghai 201203, China; 4 State Key Laboratory of Bioreactor Engineering, East China University of Science & Technology, Shanghai 200237, China; 5 State Key Laboratory of Medical Genomics, Shanghai Institute of Hematology, Rui Jin Hospital Affiliated to Shanghai Jiao Tong University School of Medicine, Shanghai 200025, China; 6 Key Laboratory of Liver and Kidney Diseases (Shanghai University of Traditional Chinese Medicine), Ministry of Education, Shanghai 200021, China

## Abstract

**Background:**

Central precocious puberty (CPP) is a common pediatric endocrine disease caused by early activation of hypothalamic-putuitary-gonadal (HPG) axis, yet the exact mechanism was poorly understood. Although there were some proofs that an altered metabolic profile was involved in CPP, interpreting the biological implications at a systematic level is still in pressing need. To gain a systematic understanding of the biological implications, this paper analyzed the CPP differential urine metabolites from a network point of view.

**Results:**

In this study, differential urine metabolites between CPP girls and age-matched normal ones were identified by LC-MS. Their basic topological parameters were calculated in the background network. The network decomposition suggested that CPP differential urine metabolites were most relevant to amino acid metabolism. Further proximity analysis of CPP differential urine metabolites and neuro-endocrine metabolites showed a close relationship between CPP metabolism and neuro-endocrine system. Then the core metabolic network of CPP was successfully constructed among all these differential urine metabolites. As can be demonstrated in the core network, abnormal aromatic amino acid metabolism might influence the activity of HPG and hypothalamic pituitary adrenal (HPA) axis. Several adjustments to the early activation of puberty in CPP girls could also be revealed by urine metabonomics.

**Conclusions:**

The present article demonstrated the ability of urine metabonomics to provide several potential metabolic clues for CPP's mechanism. It was revealed that abnormal metabolism of amino acid, especially aromatic amino acid, might have a close correlation with CPP's pathogenesis by activating HPG axis and suppressing HPA axis. Such a method of network-based analysis could also be applied to other metabonomics analysis to provide an overall perspective at a systematic level.

## Background

Central precocious puberty (CPP) is defined as the emergence of secondary sexual characteristics before the age of 8 in girls and 9 in boys due to the early activation of the hypothalamic-putuitary-gonadal (HPG) axis [1]. With an incidence of 1/5000 to 1/10000, which is higher in girls, CPP has become one of the most common pediatric endocrine diseases causing physiological and psychological difficulties for kids [2]. Physical development is a process at an overall and systematic level while the exact pathogenesis of CPP remains unknown. Some researchers found that KISS1 and GPR54 might be relevant to CPP [3,4]. There are also some proofs indicating a changed metabolic profile during puberty [5]. Recently, Jia et al. have detected a urinary metabolic signature in CPP girls by using GC/LC-MS and three pathways including catecholamine metabolic pathway, tryptophan metabolic pathway and TCA cycle were identified to be altered in CPP girls [6]. Since puberty is sensitive to metabolic cues, investigating CPP from a metabolic perspective is necessary in the way to explore its mechanism [7].

As a branch of systems biology, metabonomics or metabolomics is becoming a powerful platform providing a systematic, rapid and precise analysis of all the metabolites in biological materials [8]. Many high-throughput technologies such as GC-MS, LC-MS and NMR have been successfully used for a variety of applications including biomarker identification, drug development and disease diagnosis [9]. A general pipeline for metabonomics analysis is using the aligned spectral data combined with multivariate statistics such as PCA, OPLS or logistic regression [10]. In this way statistically different features could be selected and subsequently identified as compounds. These technologies and analysis methods have shown their power to detect a comprehensive metabolic profile [11]. Further biological understanding of metabonomics data is still waiting for systematic analysis by bioinformation technology [12].

Mapping metabolites into several distinct pathways has become a popular way in many fields including CPP research [6]. It is known that metabolites are generally organized into a complex metabolic network more than single pathways to perform their physiological function [13]. Some researchers have proposed several metabolomic correlation approaches, by which a putative metabolic network could be constructed [14,15]. There are also some researchers committing to analysis based on a real metabolic network [16]. For example, Zhao et al. found that metabolic functions were carried out in an ordered and modular way and the topological features of metabolic network could provide a functional implication [17]. These *in silico *network-based analysis methods are expected to be helpful to interpret the biological understanding if applied to metabonomics data.

Here, we analyzed 76 urinary samples from CPP girls compared to 106 controls by LC-MS. Differential urine metabolites between CPP and normal girls were identified and their basic topological parameters were calculated. A functional analysis including network decomposition and enrichment analysis was performed as well. This paper focused on analyzing the CPP's differential urine metabolites at a systematic level. The biological implication was tried to be interpreted in association with known CPP pathogenesis.

## Methods

### Subject selection and sampling

A total of 230 Chinese girls with age of 5-10 were enrolled in this study. 86 of them were diagnosed with CPP by Children's Hospital of Shanghai Jiao Tong University (Shanghai, P. R. China) and the other 144 were volunteers as age-matched healthy control. The use of these subjects was approved by the hospital's Ethics Committee and all participants provided their informed consent. Early-morning urinary samples from each individual were collected and immediately stored at -80°C after centrifugation for further analysis. Respectively, 10 and 38 samples in CPP and control group were analyzed for other reasearches. Thus 76 CPP samples and 106 healthy ones were subsequently analyzed as follow.

### Identifying differential metabolites between CPP and control

All the urinary samples were prepared and processed using the UPLC-QTOF-MS as previously described [18]. The acquired raw data files were analyzed by the MarkerLynx application manager version 4.1 for peak detection and alignment, parameters of which were set as formerly reported [18]. Peak normalization to total area for each sample was used as well. Furthermore, supervised OPLS-DA method was performed between CPP and control group to select the statistically different variations (VIP > 1) which were further validated as metabolites.

### Generating human metabolic network

Here we constructed a human global metabolic network based on KEGG database as our background network [19]. Below is a rough description of reconstruction. There are 4391 reactions that could happen in human body including 3057 enzyme-catalyzed reactions and 1334 autocatalytic ones in KEGG. The file named "reaction_mapformula.lst" in KEGG contains information of the actual direction of those reactions according to the involved pathways. In this way we excluded the currency metabolites and the reactions that unlikely happen in human body. The obtained substrates and products were afterwards connected into a directed network which contained 3114 nodes and 4642 arcs.

### Topological parameters

In a network, centralities of a node, including degree, betweeness and closeness, are used to measure its contribution to the communication between other nodes. Connectivity and distance, both belonging to proximities, are designed to measure how closely the nodes are connected and how far they are away from each other, respectively. All these parameters were introduced in this paper to explore the functional characteristics of CPP's differential metabolites in global metabolic network (see Additional file 1).

### Significant level

#### Z-score

Z-score (see Additional file 1) is designed to evaluate whether a topological feature of CPP differential urinary metabolites is significantly different from its corresponding randomizations. Generally, a topological feature is accepted to have a statistical significance if |*Z*| > 2.33.

#### P-value

In this paper, p-value is defined by the hypergeometric cumulative distribution function (see Additional file 1). It represents the chance that at least *k *CPP metabolites co-exist in the same module, cluster or pathway. A cutoff of P < 0.05 means that a pathway or cluster is enriched by CPP differential urine metabolites.

### Network decomposition

Aiming to divide the whole graph into several functional areas, the constructed human metabolic network was broken up by simulated annealing algorithm, which helps to get a nearly best decomposition with the maximum modularity [20,21]. The most connected part of the background network was extracted by removing the isolated subset (IS) of the bow-tie structure. Then it was decomposed into several modules, which were subsequently expanded to larger modules respectively. There are a few rules for expanding: (i) a node will be assigned to the module which it directly connected with; (ii) if a node is attached to several modules, it will be assigned to the module with more nodes directly connected with it; (iii) this process ends when there are no more nodes that could be added to any module.

Considering that the obtained modules may fail to have a distinct function and the CPP differential urine metabolites also might be too much distracted, a step of clustering was followed. This step of clustering was processed by Ward algorithm performed in R based on the Euclidean distances between any two modules (*E_i, j_*, see Additional file 1) [22].

## Results and discussion

### Centralities of CPP differential urine metabolites

Totally, 99 distinct CPP differential urine metabolites were obtained by comparing CPP and normal girls' metabolic profiles identified by LC-MS. To explore these metabolites' internal connection, we mapped them in the global human metabolic network. Among the 99, 71 were annotated by KEGG database, and 49 were connected in our constructed global human metabolic network. The centralities of these 49 metabolites were investigated in the background network.

To evaluated their importance in the network context, degrees, betweeness and closeness of these metabolites were computed by Pajek, a software designed for complex graph [23]. As control, randomizations were generated by randomly selecting 49 nodes from the background network for 10^5 ^times. In- and out- degree or closeness were computed respectively because the background network was a direct graph. The centralities of the 49 CPP differential urine metabolites were listed in Table 1.

**Table 1:** Centralities of CPP differential urine metabolites.

Parameters	human_meta_net	CPP_compounds	Random_everage	Random_sd	Z-score
In-degree	1.49	2.27	1.49	0.17	4.50
Out-degree	1.49	2.37	1.49	0.21	4.21
Closeness_in	0.0076	0.0122	0.0076	0.0014	3.27
Closeness_out	0.0076	0.0141	0.0076	0.0016	4.17
Betweeness	0.0004	0.0016	0.0004	0.00025	4.71

As can be seen in Table 1, CPP differential urine metabolites were shown to have significantly higher degree, betweeness and closeness comparing to the corresponding randomizations (|*Z*| > 2.33). As the degree of a node measures how many other nodes it may connect with, the higher in- and out- degree shown in Table 1 indicates that CPP differential metabolites tend to be located in the hubs of the network. This was subsequently confirmed by the following bow-tie structure analysis that revealed CPP metabolites were enriched in the giant strong component (see Additional file 2) which was the most connected part in the network. Closeness (in or out) is a metric representing the independence and efficiency of a node in communication. And betweeness represents the potential of a node for controlling information exchanging in the network. The higher closeness and betweeness shown in Table 1 suggested that those CPP differential metabolites may be critical to the communication between other metabolites in the network. Overall, these parameters of centrality including degree, closeness and betweeness all indicated an important role of CPP differential urine metabolites in the human metabolic network.

### Enriched functional modules

To interpret the biological functions of CPP's metabolic clues, the enriched functions of CPP differential urine metabolites were analyzed in the background of human metabolic network extracted from KEGG. Two aspects were involved: primary metabolism and neuro-endocrine system. The former focused on primary metabolic function and the latter investigated the physiological function in CPP.

#### Enriched primary metabolic module

**Figure 1:**
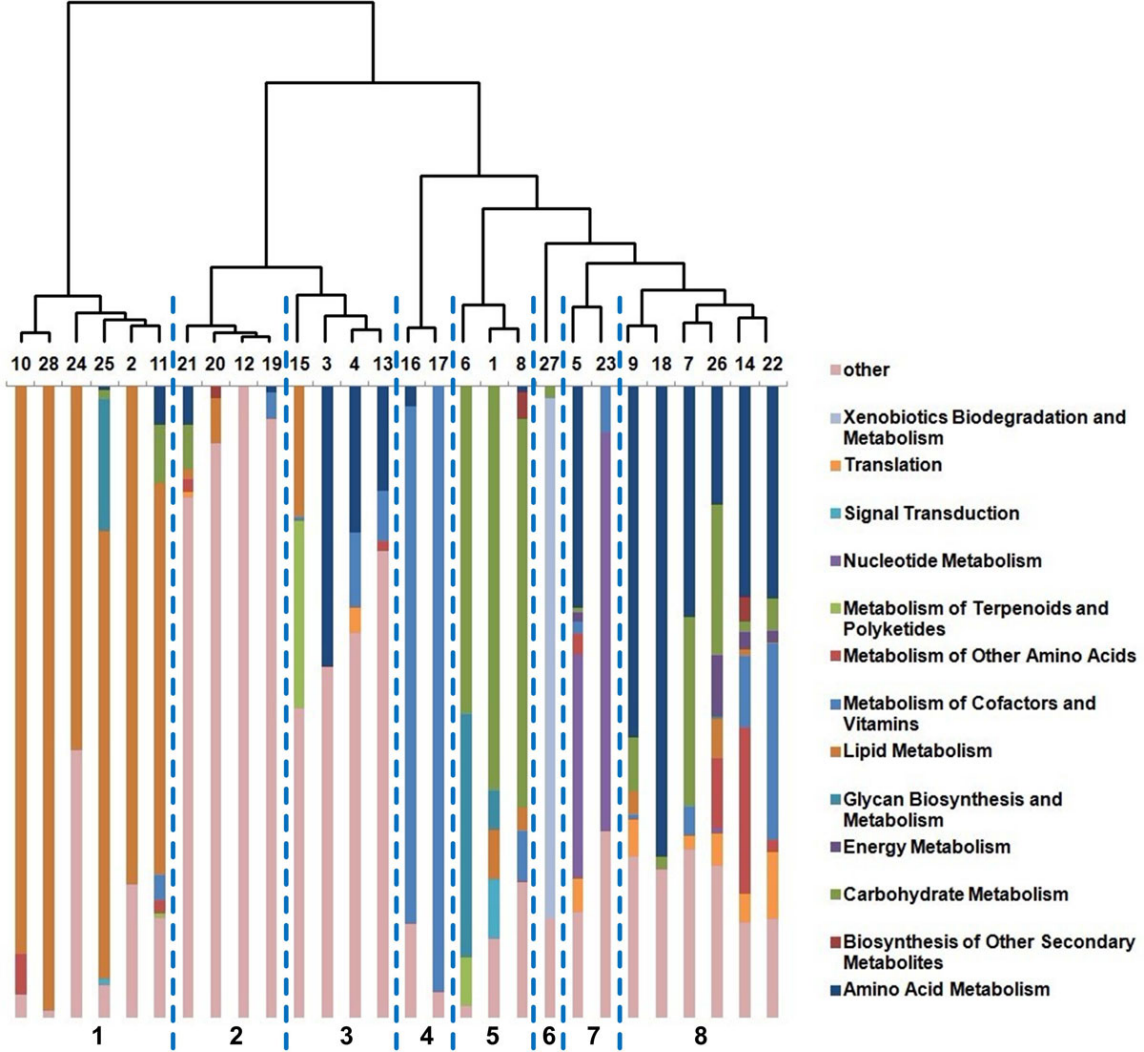
**Hierarchical clustering for modules**. Each rectangle represents a module and the number above is the module's index. Each color denotes a biological process according to the KEGG pathway classification and its area in one rectangle reflects the proportion of the corresponding biological process in this module. The biological processes named "other" are reactions that do not exist in any KEGG human pathway. The dotted lines classified the 28 modules into 8 clusters according to the hierarchical tree in this figure.

By using a classic simulated annealing algorithm and expanding, the background network was decomposed into 28 modules (modularity = 0.886957) which consisted of 1386 nodes [20,21]. 32 CPP differential urine metabolites were included in these modules while the others were excluded as isolated nodes. This decomposition has a significantly higher modularity (Z = 42.53) than the 50 corresponding random decompositions. The proportions of differential biological processes in each module were computed. Then the euclidean distance between different modules was calculated and Ward algorithm was applied for clustering. All modules and their contained biological processes were shown in Figure 1 in a clustering representation. As can be seen in Figure 1, these modules could be classified into 8 clusters according to Ward algorithm. The main functions of these clusters were listed in Table 2, which covered metabolism function of various compounds such as amino acids, nucleotides, xenobiotics, carbohydrate, vitamins and lipids. In Table 2, it is interesting to see that CPP's differential urine metabolites are found to be only enriched in cluster 8 (P < 0.05) whose main function is amino acid metabolism, suggesting that amino acid metabolism may be mostly related to CPP compared to other types of metabolism from the current urine metabonomics data. Since this is an overall reflection of aberrant urine metabolites, the exact correlation between CPP and amino acid metabolism deserves further investigation.

**Table 2:** Main function of each cluster and corresponding enrichment of CPP differential urine metabolites.

Cluster ID	Main Function	Modules included	Total metabolites	CPP differential urine metabolites	P-value
1	Lipid metabolism	10,28,24,25,2,11	280	3	0.971
2	Unknown	21,20,12,19	170	1	0.986
3	Largely unknown containing partial amino acid metabolism	15,3,4,13	251	4	0.859
4	Metabolism of Cofactors and Vitamins	16,17	51	1	0.703
5	Carbohydrate Metabolism	6,1,8	165	4	0.541
6	Xenobiotics Biodegradation and Metabolism	27	33	0	1.000
7	Nucleotide Metabolism	5,23	152	7	0.053
8	Amino Acid Metabolism	9,18,7,26,14,22	284	12	0.019

#### Correlation with neuro-endocrine system

CPP is widely accepted as an endocrine disorder caused by early activation of HPG axis which belongs to the neuro-endocrine-immune (NEI) system. Thus whether the CPP differential urine metabolites may correlate with the NEI system was examined by calculating their proximity. NEI interaction has been acknowledged as the main regulatory component in host's homeostasis since it was put forward in 1977 [24]. Database of dbNEI has provided a molecular resource ranging from compounds, peptides to proteins for the NEI system [25,26]. A total of 356 KEGG-annotated metabolites were collected from dbNEI and their proximity to those CPP metabolites was calculated in the background network. Since there was no immune metabolite in our background network, only the proximity between metabolites involved in CPP and neuro-endocrine system could be computed. As control for CPP, 10^5 ^randomizations were generated by randomly selecting 49 nodes from the background network. CPP differential urine metabolites were found to have a higher connectivity and lower distance to neuro-endocrine metabolites than the commensurable randomizations (both |*Z*| > 2.33, see Additional file 3). This suggested that CPP differential urine metabolites tended to have a more tight connection to neuro-endocrine system comparing to the random control.

**Figure 2:**
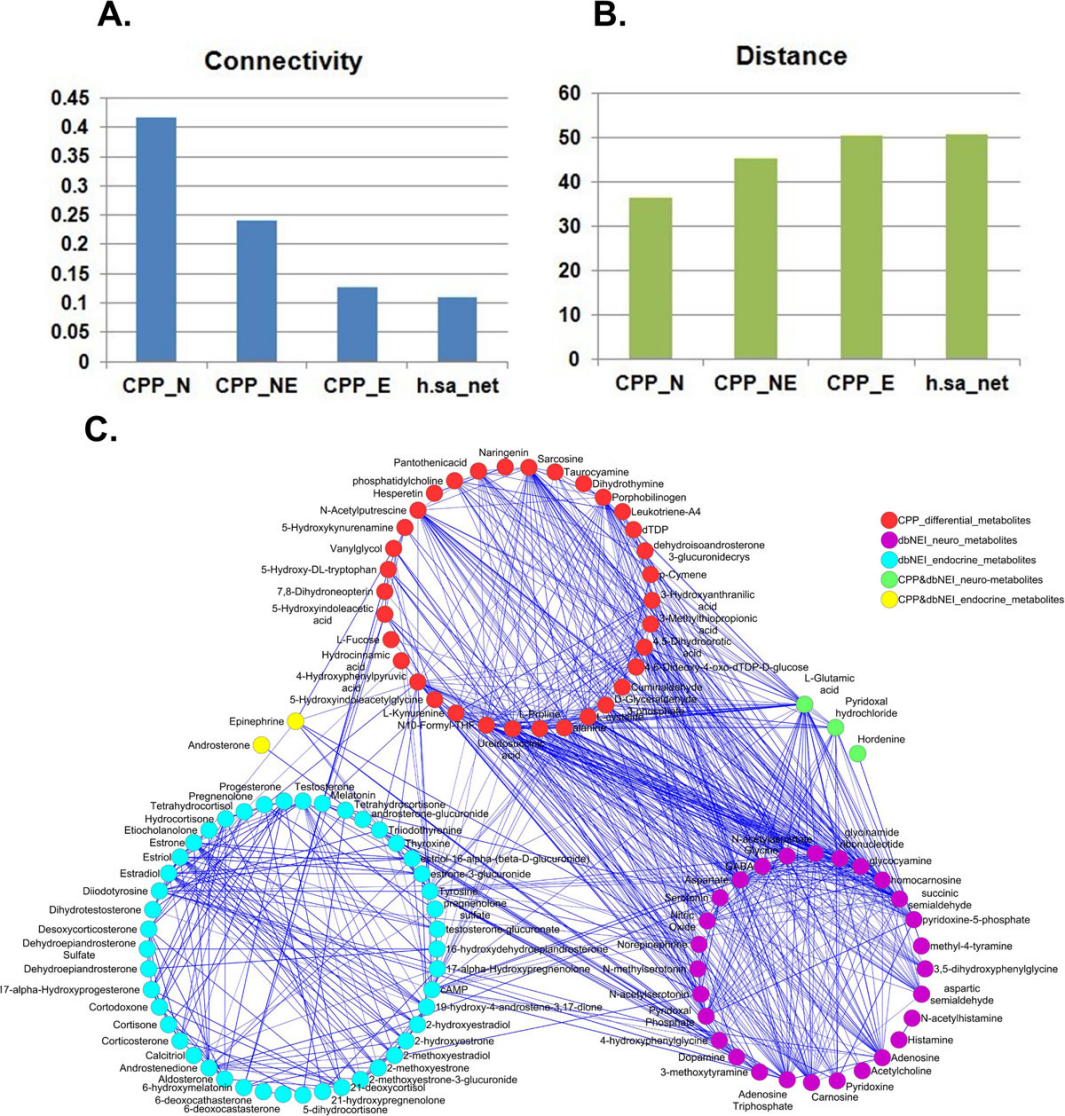
**Correlation between CPP differential urine metabolites and neuro-endocrine metabolites**. A. Connectivity between CPP differential urine metabolites and neuro-endocrine metabolites. B. Distance between CPP differential urine metabolites and neuro-endocrine metabolites. C. Simplified subnet of CPP differential urine metabolites and neuro-endocrine metabolites. Abbreviations: CPP_N, CPP and neuro-system; CPP_E, CPP and endocrine-system; CPP_NE, CPP and the whole neuro-endocrine system; h.sa_net, the global human metabolic network. There was no CPP_I (CPP and immune system) because no immune metabolite was connected in the background network.

Furthermore, the connectivity and distance of CPP metabolites and neuro-endocrine metabolites were shown as histogram in Figure 2A and 2B, respectively. Meanwhile, for visualization, a simplified sub-network which contains metabolites from CPP and neuro-endocrine system was extracted as shown in Figure 2C. In Figure 2, it can be noticed that the proximity of CPP to nervous system was closer than that to the endocrine system not only in statistics (2A and 2B) but also in visualization (2C). It was indicated that CPP differential urine metabolites may be more correlated with the activities of nervous system than endocrine system. For instance, glutamate is a well-known excitatory amino acid in the central nervous system (CNS) and could directly mediate synaptic neurotransmission [27]. It was detected to be down-regulated in CPP urine samples. Thus the differential urine metabolites of CPP girls may help to provide a metabolic clue to the early activation of HPG axis in euro-endocrine system.

### Core metabolic network of CPP

#### Constructing the core network of CPP differential urine metabolites

To construct a minimized core network for CPP differential urine metabolites, enriched pathways were firstly screened for integration. Totally, 7 pathways were significantly enriched by CPP differential urine metabolites. Table 3 is a rank of these pathways according to their p-value. In Table 3, among the 7 ranked pathways, tryptophan metabolism (1st) and tyrosine metabolism (6th) were also observed by Jia et al. although based on different analytical methods [6]. In this sense, the reliability of these candidate pathways can be supported. Besides, pyrimidine metabolism (5th) was picked up as well which agreed with previous enrichment data in nucleotide metabolism (cluster 7 in Table 2). Interestingly, as also can be seen from Table 3, 6 among 7 enriched pathways were metabolisms of amino acid. This observation supported the above finding in network decomposition. The detailed correlation between CPP and amino acid metabolism would be further interpreted as follow.

**Table 3:** Enriched pathways of CPP differential urine metabolites

Rank	pathway	Total metabolites in pathway	CPP differential urine metabolites in pathway	P-value
1	Tryptophan metabolism	81	8	0.00039
2	Taurine and hypotaurine metabolism	20	3	0.003942
3	Alanine, aspartate and glutamate metabolism	24	3	0.006682
4	Aminoacyl-tRNA biosynthesis	75	5	0.007183
5	Pyrimidine metabolism	59	4	0.015266
6	Tyrosine metabolism	76	4	0.035101
7	Arginine and proline metabolism	84	4	0.047985

**Figure 3:**
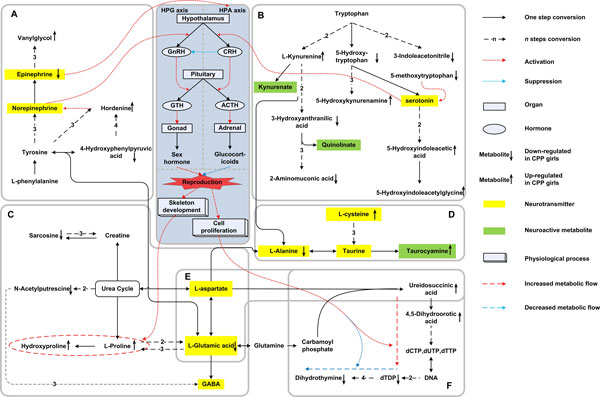
**Core network of CPP differential urine metabolites**. Gray boxes represent distinct pathways: (A) Tyrosine metabolism; (B) Tryptophan metabolism; (C) Arginine and proline metabolism; (D) Taurine and hypotaurine metabolism; (E) Alanine, aspartate and glutamate metabolism; (F) Pyrimidine metabolism. Abbreviations: HPG, hypothalamic pituitary gonadal; HPA, hypothalamic pituitary adrenal; CRH, corticotropin releasing hormone; GnRH, gonadotropin releasing hormone; ACTH, adrenocorticotropic hormone; GTH, gonadotropic hormone.

As CPP was reported to be mainly regulated by HPG and hypothalamic pituitary adrenal (HPA) axis, HPG and HPA axis can be regarded as an initial center for the core network construction [28]. The above 7 enriched pathways were assigned around and integrated into a core metabolic network in form of a comprehensive graph as displayed in Figure 3. In Figure 3, the minimized core metabolic network of CPP contained a total of 40 compounds including 24 CPP differential urine metabolites. The box in the center highlighted in blue represents HPG and HPA axis and gray boxes were used to separate distinct pathways. All the CPP differential urine metabolites were followed by an up- or down- arrow respectively denoting up- or down- regulated in CPP group. Neurotransmitters and neuroactive metabolites were respectively highlighted in yellow and green. An information flow passing through the HPG and HPA axis could be observed in Figure 3. The upstream was aromatic amino acid metabolism and the downstream was primarily proline and pyrimidine metabolism. Following part is a detailed discussion.

#### Perturbation of Neurotransmitters and Neuroactive Metabolites

As can be found in Figure 3, although the network was constructed mainly based on CPP unrine metabolites, many compounds (12 among 40) were neurotransmitters or neuroactive metabolites. The 9 neurotransmitters highlighted in yellow include epinephrine, norepinephrine, serontonin, cysteine, taurine, alanine, glutamic acid, aspartate and GABA. And four (4/9) of them were found to be differentially expressed in CPP and the others were all closely related to the CPP differential urine metabolites. A similar situation also exists in the 3 neuroactive metabolites, as being highlighted in green. One of them, taurocyamine, a inhibitor for taurine transportion and also a antagonist for glycine receptor, was directly interfered in CPP [29]. The other two, kynurenate and quinilnate, were one- or two- step away from CPP differential metabolites. Kynurenate is an extensive antagonist for excitatory amino acid receptors [30], and quinilnate has been reported with a potential toxicity in some neurodegenetative diseases [31]. Despite of the observation, further CPP pathogenesis and the relationship between cause and effect might be worthy of validation.

#### Upstream of HPG axis and HPA axis

As Figure 3 shows, upstream of the HPG and HPA axis is the metabolism of aromatic amino acids. There were two main pathways: tyrosine metabolism and tryptophan metabolism.

##### Tyrosine metabolism

This pathway was shown in box A of Figure 3. It has been reported that epinephrine can stimulate the activation of HPA axis while the HPA axis could inhibit female reproduction at multi-levels [32,33]. As can be seen in Box A, epinephrine was found to be down-regulated in CPP patients, indicating a decreased activation of HPA axis and further switching on the initiation of female reproduction. By the way, HPA axis in females was reported to be more sensitive than that in males [34]. This could be one of the reasons why girls are more incident to CPP than boys.

Secondly, hordenine could accelerate the secretion of norepinephrine [35]. Then norepinephrine could facilitate hypothalamic cells to secrete gonadotropin-releasing hormone (GnRH) which could directly activate the HPG axis and subsequently promote the development of reproduction system [34]. In this study, hordenine was detected to be up-regulated in the CPP urine samples and may finally promote the sex hormone secretion with norepinephrine as a media.

Furthermore, vanylglycol can be regarded as a reflection of the excitement of the noradrenergic neurons in CNS and sympathetic nervous system [36]. The significant up-regulation of vanylglycol in CPP urine group might suggest a more active state of patients' neuro-endocrine system than normal ones.

##### Tryptophan metabolism

This pathway was represented in box B of Figure 3. In this pathway, serotonin could suppress HPG axis and activate HPA axis by exciting the corticotrophin-releasing hormone (CRH) neuron [32]. Although serotonin was not directly detected as CPP differential metabolites in this study, several related metabolites could provide some clues. In CPP girls' urine samples, the precursor of serotonin, 5-hydroxy-tryptophan, was detected to be down-regulated while the terminal products of serotonin, 5-hydroxyindoleacetic acid and 5-hydroxyindoleacetylglycine, was recognized to be up-regulated. But this evidence was not strong enough to infer serotonin's level in CPP girls. More directly, the concentration of 5-methoxytryptophan (ML) was reported to be consistent with serotonin in human body and ML was identified to be down-regulated in CPP group in this study [37]. The diminished ML may imply a lower level of serotonin and further contribute to the activation of HPG axis and suppression of HPA axis.

#### Downstream of HPG axis and HPA axis

As Figure 3 shows, downstream of the HPG and HPA axis is mainly proline metabolism and pyrimidine metabolism. Proline and hydroxyproline are essential components for collagen. In box C of Figure 3, the high urine concentrations of proline and hydroxyproline in CPP girls could indicate the need for skeleton development during puberty. In pyrimidine catabolism (box F), DNA synthesis was enhanced whereas DNA degradation was weakened. A resulting accumulated amount of DNA could be used for the cell proliferation in the basic development in CPP girls. There were also some other adjustments to the precocious puberty. For example, epinephrine can not only regulate the nervous system as a neurotransmitter but also stimulate the lipolysis [38]. Accordingly, the CPP's lower level of epinephrine might contribute to the higher body mass index (BWI) of CPP girls (p ≪ 0.01, one-tailed Wilcoxon test).

## Conclusions

In this study, the potential metabolic mechanism of CPP was tried to be interpreted by a network analysis method to the urine metabonomics data at a systematic level. A core network of CPP differential urine metabolites was also generated as a comprehensive graph. Our results demonstrated that (i) Abnormal amino acid metabolism might be most relevant to CPP based on the urine metabolite profile; (ii) The urine metabolic profile of CPP girls could reflect the abnormal activity of neuro-endocrine system; (iii) Specifically, aromatic amino acid metabolism might contribute to CPP pathogenesis by activating HPG axis and suppressing HPA axis; and (iv) Several adjustments to the early activation of puberty in CPP girls could also be revealed by urine metabonomics. The network analysis method in this study could also be applied to further biological implications interpreting from metabonomics data by providing an overall perspectives at the systematic level.

## Competing interests

The authors declare that they have no competing interests.

## Authors' contributions

LY and KT conceived this research, designed the analysis, and prepared the primary manuscript. YQ performed the sample preparation and data acquisition; HY assisted in data analysis; WC participated in the design of the study; YZ and ZC supervised the project and edited the manuscript. All authors read and approved the final manuscript.

## Supplementary Material

Additional file 1 **Supplementary methods**. A detailed description of topological parameters, significant level and Euclidian distance involved in this paper. Click here for file

Additional file 2 **Table S1**. Enrichment of CPP differential urine metabolites in the bow-tie structure of human metabolic network. Click here for file

Additional file 3 **Table S2**. CPP differential urine metabolites' proximity to neuro-endocrine system. Click here for file
