# Supplementary material for: Potential metabolic mechanism of girls' central precocious puberty: a network analysis on urine metabonomics data
Source: BMC Syst Biol. 2012 Dec 17;6(Suppl 3):S19. doi: 10.1186/1752-0509-6-S3-S19 (PMC3524310; doi:10.1186/1752-0509-6-S3-S19)
Supplement: Additional file 1 — Supplementary methods. A detailed description of topological parameters, significant level and Euclidian distance involved in this paper. [file 1752-0509-6-S3-S19-S1.docx]

# Additional file 1

# Topological parameters of network

## Centrality

***Degree.*** In a directed network, there are two kinds of degree: in-degree and out-degree. The in-degree of a node is the number of arcs adjacent to it with head endpoints, and the number of arcs with tail endpoints is its out-degree.

***Betweeness.*** Betweeness is the probability of a node (*i*) occurring on a shortest path between other nodes. It represents the potential of a node (*i*) for controlling information exchanging in the network. The betweeness of a node (*i*) could be defined by

$$Betweeness(P_{i})= \frac{2N_{i}}{n^{2}-3n+2}$$

where *N_i_* is the total number of the shortest paths passing the node *i*, and *n* is the total number of nodes in the network.

***Closeness.*** Closeness is a metric representing the independence and efficiency of a node in communication. It can be calculated as

$$Closeness\left( P_{i} \right)= \frac{n-1}{\sum_{j=1}^{n} N(j, i)}$$

where *n* is the total number of nodes and *N(j, i)* is the length of the shortest path between the node *i* and the node *j*.

## Proximity

***Connectivity.*** In graph theory, two nodes can be called connected if at least one path exists between them, otherwise unconnected. Here we use the connectivity *C_I,J_* to measure how closely two groups of nodes (*I* and *J*) are connected. The equation is

$$C_{I, J}= \frac{\sum_{i} \sum_{j} c_{i,j}}{N_{I}N_{J}}$$

*c _i,j_* is an integer which equates 1 if *i* and *j* are connected, 0 or else. *N_I_* and *N_J_* are the total number of nodes in group *I* and group *J*, respectively.

***Distance.*** In a network, the distance of two distinct nodes is the length of the shortest path between them. Hence, the distance (*D_I,J_*) between two groups of nodes (*I，J*) is defined as follows.

$$D_{I, J}= \frac{\sum_{i} \sum_{j} d_{i,j}}{N_{I}N_{J}}$$

*d _i,j_* is the distance between node *i* and node *j*. *N_I_* and *N_J_* are the total number of nodes in group *I* and group *J*, respectively.

# Significant level

## Z-score

Z-score measures whether a feature is significantly different from the corresponding randomizations which are generated by randomly selecting *k* nodes from the background network for 10^5^ times, where *k* is an integer equalling to the number of CPP differential urine metabolites in network. The equation is:

$$Z= \frac{\overline{P}-\overline{P}_{r}}{\Delta P_{r}}$$

$\overline{P}$: the average value of a topological feature of CPP metabolites.

$\overline{P}_{r}$: the average value of a topological feature of 100,000 randomizations.

$\Delta P_{r}$: the standard deviation of a topological feature of 100,000 randomizations.

Generally, a topological feature has a statistical significance if $\left| \left. Z \right| \right.$>2.33.

## P-value

In this article, p-value is defined by a hypergeometric cumulative distribution function to represent the probability of gathering at least *k* desired samples in a group out of a population by chance. It can be calculated by the formula below.

$$P-value=1- \sum_{i=0}^{K-1} \frac{C_{K}^{i}\cdot C_{N-K}^{k-i}}{C_{N}^{k}}$$

*N*: total number of samples in the sampling population.

*K*: total number of desired samples in the sampling population.

*k*: number of samples in one group.

*i*: number of desired samples in one group.

# Euclidean distance

The global human metabolic network can be decomposed into several modules by simulated annealing algorithm. Considering that the obtained modules are small units and the CPP differential urine metabolites also might be too much distracted, a step of clustering was necessary. This step of clustering was processed by Ward algorithm based on the Euclidean distances between any two modules (*E_i,j_*). Below is a detailed description of the calculation of Euclidean distances.

(i) In our background network, each arc might belong to several pathways. According to KEGG, pathways can be assigned into several biological processes. To evaluate a biological process’s proportion in a module, each arc should be weighted according to the number of pathways they involved in. There are two kinds of arcs in the decomposed network: arcs within a module and arcs between two modules. For arcs within a module, the weight can be calculated by the equation:

$$w_{a}= \frac{1}{N_{a}}$$

For arcs between two modules, the weight can be calculated by the equation:

$$w_{a}= \frac{1}{{2N}_{a}}$$

Where $w_{a}$ is the weight of arc *a*, and $N_{a}$ is the the number of pathways that arc *a* involved in.

(ii) Then the weight of an arc can be normalized by the following equation:

$${w‘}_{a}=\frac{w_{a}}{\sum_{a\in A} w_{a}}$$

Where $w_{a}$ is the weight of arc *a*, and ${w'}_{a}$ is the normalized weight of arc *a*, A is the set of all arcs in the decomposed network.

(iii) Furthermore, a biological process’s proportion in a module can be computed as follow:

$$P_{m, l}= \frac{\sum_{a\in(L\bigcap M)} w_{a}}{\sum_{a\in M} w_{a}}$$

$P_{m, l}$: proportion of biological process *l* in module *m*.

$L$: a set of arcs involving in biological process *l.*

$M$: a set of arcs involving in module *m.*

(iv) Based on the computed $P_{m, l}$, Euclidean distance between any two modules ($E_{i, j}$) were subsequently calculated and formed into a matrix which was prepared for further clustering. $E_{i, j}$ can be calculated by the equation below:

$$E_{i, j}=\sqrt{\sum_{l}^{L} {(P_{i, l}-P_{j, l})}^{2}}$$

*E_i, j_*: Euclidean distance between module *i* and module *j*.

*P_i, l_*: proportion of biological process *l* in module *i*.

*P_j, l_*: proportion of biological process *l* in module *j*.

*L*: all the biological processes involved in all modules.

Finally, the obtained $E_{i, j}$ can form into a distance matrix and subsequently processed by R for further clustering.
