# Supplementary material for: Potential metabolic mechanism of girls' central precocious puberty: a network analysis on urine metabonomics data
Source: BMC Syst Biol. 2012 Dec 17;6(Suppl 3):S19. doi: 10.1186/1752-0509-6-S3-S19 (PMC3524310; doi:10.1186/1752-0509-6-S3-S19)
Supplement: Additional file 2 — Table S1. Enrichment of CPP differential urine metabolites in the bow-tie structure of human metabolic network. [file 1752-0509-6-S3-S19-S2.docx]

# Additional file 2

## Table S1. Enrichment of CPP differential urine metabolites in the bow-tie structure of human metabolic network

| Part of bow-tie* | CPP differential metabolites | Total metabolites | P-value |
| --- | --- | --- | --- |
| GSC | 22 | 725 | 6.34E-04 |
| S | 4 | 191 | 0.35 |
| P | 4 | 323 | 0.76 |
| IS | 19 | 1875 | 1 |
| Total | 50 | 3114 |  |

*There are four parts in the bow-tie structure of a network: giant strong component (GSC), substrate subset (S), product subset (P) and isolated subset (IS).
