# Supplementary material for: Potential metabolic mechanism of girls' central precocious puberty: a network analysis on urine metabonomics data
Source: BMC Syst Biol. 2012 Dec 17;6(Suppl 3):S19. doi: 10.1186/1752-0509-6-S3-S19 (PMC3524310; doi:10.1186/1752-0509-6-S3-S19)
Supplement: Additional file 3 — Table S2. CPP differential urine metabolites' proximity to neuro-endocrine system. [file 1752-0509-6-S3-S19-S3.docx]

# Additional file 3

## Table S2. CPP differential urine metabolites’ proximity to neuro-endocrine system

|  | **Connectivity** | | | |  | **Distance** | | | |
| --- | --- | --- | --- | --- | --- | --- | --- | --- | --- |
|  | Mean | Random_mean** | Random_sd | Z-score |  | Mean | Random_mean | Random_sd | Z-score |
| CPP_N* | 0.417 | 0.257 | 0.045 | 3.520 |  | 36.518 | 44.024 | 1.947 | -3.855 |
| CPP_E | 0.127 | 0.082 | 0.014 | 3.235 |  | 50.399 | 52.022 | 0.522 | -3.111 |
| CPP_NE | 0.242 | 0.152 | 0.026 | 3.452 |  | 45.151 | 48.859 | 1.065 | -3.480 |

*CPP_N, CPP and neuro-system; CPP_E, CPP and endocrine- system; CPP_NE, CPP and the whole neuro-endocrine system.

**The randomization was generated by randomly selecting 49 nodes from the background network for 10^5^ times.
